# Research on Improving Online Purchase Intention of Poverty-Alleviation Agricultural Products in China: From the Perspective of Institution-Based Trust

**DOI:** 10.3389/fpsyg.2022.900328

**Published:** 2022-05-12

**Authors:** Guangming Li, Xianghua Wu, Chao Yuan

**Affiliations:** ^1^Business School, Hohai University, Nanjing, China; ^2^The School of Information and Library Science, The University of North Carolina at Chapel Hill, Chapel Hill, NC, United States

**Keywords:** poverty-alleviation by consumption, online purchase intention of poverty-alleviation agricultural products, institution-based trust, helping the poor, trust

## Abstract

Poverty alleviation by consumption is a powerful way to help the poor people get rid of poverty, which plays a significant role in China's rural revitalization. However, the achievement of poverty alleviation by consumption mostly depends on government procurement, and the enthusiasm of customers to participate is low, facing the severe challenge of poor sustainability. Helping the poor is the most common motivation for customers to buy poverty-alleviation agricultural products (PAAP). However, as the negative events of poverty alleviation such as “tragic marketing” constantly appear in news reports, customers' trust in sellers has been seriously damaged. The psychological protection system for fear of being cheated hinders customers' purchase intention. Therefore, we believed that trust is an important factor in enhancing customers' purchase intention of PAAP. Customers buy PAAP mainly through online channels, and institution-based trust is the most important way to generate trust in online channels. Thus, this study investigated the institutional mechanisms that affect customers' trust in the sellers of PAAP and discussed the influence of trust on the online purchase intention of PAAP. Data were obtained through a questionnaire survey and tested empirically. The results showed that the effectiveness of the user feedback mechanism, platform supervision mechanism, product traceability mechanism, and product certification mechanism can enhance customers' purchase intention by enhancing their trust. Individual trust tendency positively regulated the relationship between the effectiveness of institutional mechanisms and consumer trust. The conclusion can not only provide new theoretical guidance for the practice of poverty alleviation by consumption in China but also offer new ideas for the poverty alleviation undertakings in other countries.

## Introduction

In 2021, the No. 1 Document of the China Central Committee pointed out that China is now in a smooth transition period from poverty alleviation to the comprehensive promotion of rural revitalization. It should continue to promote rural revitalization in poverty-stricken areas and expand poverty alleviation by consumption^1^ Poverty alleviation by consumption is a new type of poverty-alleviation method in which people from all walks of life consume products and services from poor people to help them increase income and get rid of poverty (Li, 2021). Statistics show that the sales of poverty-alleviation products in China reached 306.94 billion CNY (US$48.235 billion) in 2020, effectively boosting the income of the poor and lifting them out of poverty^2^ However, while achieving gratifying achievements, poverty alleviation by consumption also has some problems that cannot be ignored, among which the most serious is the sustainability of poverty alleviation by consumption. Currently, the participants of poverty alleviation by consumption are mainly government departments such as parties and government organs at all levels and state-owned enterprises and institutions, and the enthusiasm of customers to participate in purchasing is low (Fan and Liu, 2021). According to data released by the National Bureau of Statistics, the total retail sales of consumer goods in China in 2020 was 3.981 trillion CNY ($6,256.09 billion), and the sales of poverty-alleviation products accounted for only 0.78%. In other words, < 1 CNY (US$0.15) is spent on poverty-alleviation products for every 100 CNY (US$15.72) of household consumption. This mode of assistance, which relies mainly on government procurement and lacks a long-term guarantee mechanism, is not only detrimental to the sustainable development of poverty alleviation by consumption, but also seriously hinders the promotion of rural revitalization, and is in urgent need of adjustment and optimization (Quan, 2021). Electronic commerce platforms are the most important channels for customers to purchase poverty-alleviation products, and their online shopping for poverty-alleviation products mainly focuses on agricultural products. Therefore, it is of great significance to study how to enhance customers' willingness to buy poverty-alleviation agricultural products (PAAP) online.

As a unique poverty-alleviation method in China, the related research on poverty alleviation by consumption is mainly concentrated in China and focuses on its connotation, significance, and policy measures (Chen, 2019; Sun and Wang, 2020; Liu and Liao, 2021; Liu et al., 2021), but seldom on the purchase intention of PAAP. Few studies related to purchasing intention of PAAP mainly paid attention to the influence of quality characteristics of PAAP on purchase intention (Feng et al., 2021). For example, Fan and Liu (2021) thought that there were some problems in PAAP, such as single structure, poor product quality, and low brand awareness, which restricted customers' purchase intention. Li and Ning (2019) also pointed out that, in recent years, Chinese customers' demand for agricultural products has changed from quantity to quality. Their consumption trend is characterized by grading, individualization, and diversity, but the poverty-stricken areas still maintain the traditional, relatively single and backward agricultural production structure. The contradiction between supply and demand seriously limits the sales of PAAP. Long et al. (2021) found that agricultural products provided by poor areas in China have certain problems such as “production without quantity,” “quantity without product,” “product without quality,” “quality without sound,” and so on, which are out of line with the high standard and high-quality demand of customers in urban areas. The mismatch between supply and demand severely restricts urban customers' willingness to buy PAAP (Yuan, 2020).

However, current research has not focused on the role of the poverty-alleviation function attribute of PAAP in improving customers' purchase intention. As poverty alleviation by consumption has dual meanings of “consumption” and “poverty alleviation,” customers' motivation for purchasing PAAP should be a combination of the private interest motivation in traditional economics and the public welfare motivation in charity economics. The investigation of Zeng et al. (2021) showed that helping the poor is the most common motivation for customers to buy PAAP (Zeng et al., 2021). In the initial stage of purchasing, most customers focus on the poverty-alleviation function of PAAP. They care about whether PAAP can really benefit the poor. Currently, China's poverty alleviation by consumption is still in its infancy, and, in the market development of PAAP, customers driven by poverty-alleviation function attributes will become the main purchasing force. E-commerce platforms such as Pinduoduo, JD.COM, and Suning.cn are the main channels for them to purchase PAAP. However, customers' trust in sellers has been seriously damaged after the negative events such as “slow sales,” “false propaganda,” and “posturing” in news reports. The lack of trust seriously restricts customers' online purchase intention of PAAP (Zhou, 2020). Previous studies have pointed out that the most important way to generate trust in online sellers is to make customers feel that transactions are protected by institutional mechanisms (Zucker, 1986). Then, which institutional mechanisms will affect customers' trust in the sellers of PAAP? Can trust significantly enhance customers' willingness to purchase PAAP online? To motivate the enthusiasm of customers to buy PAAP online and thus provide the impetus for sustainable development of poverty alleviation by consumption, this study investigated the institutional mechanisms that affect customers' trust in the sellers of PAAP and discussed the influence of trust on online purchase willingness of PAAP. The conclusion can not only provide new theoretical guidance for the practice of poverty alleviation by consumption in China but also offer new ideas for the poverty alleviation undertakings in other countries.

## Theoretical Basis and Research Hypothesis

### Theoretical Basis

(1) Connotation and characteristics of PAAP

Poverty-alleviation agricultural products refer to agricultural products with poverty-alleviation effects recognized by the National Rural Development Bureau, which are produced in the poor areas of central and western China. The function of PAAP is “to make the policy dividend and market dividend of poverty alleviation by consumption finally fall on industries in poverty-alleviation areas, and to drive the poor people to increase their incomes and get rid of poverty.”

(2) Institution-based trust and institutional mechanisms

Trust refers to one party's expectation that the other party will not engage in opportunistic behavior (Wei et al., 2019). Customers' trust in the sellers of PAAP (referred to as customers' trust) means that customers do not have opportunistic expectations of the sellers of PAAP, believing that sellers will transact as expected and sell genuine and qualified PAAP. Zucker (1986) pointed out that there are many ways to generate trusts, such as cognition-based trust, knowledge-based trust, calculative-based trust, and institution-based trust. Among them, institution-based trust is the most important trust creation model in the business environment, which is generally agreed upon by e-commerce researchers (Bruckes et al., 2019; Wei et al., 2019). When customers believe that reliable institutional mechanisms already exist, which can provide a guarantee for online transactions and online sellers will trade in a trustworthy way, they will have trust in online sellers (Yuliati et al., 2020; Guo et al., 2021).

The institution consists of a series of regulations, rules, or constraints that affect human behavior. Institutional mechanisms are commitments, regulations, legal resources, or other procedures established and implemented by third-party organizations to ensure the proper conditions under which transactions take place. These mechanisms are institutionalized to ensure that all transactions in complex trading situations can be carried out in a set way (He and Liang, 2018). In the field of e-commerce, the institutional mechanisms that affect customers' trust include authentication mechanisms, hosting mechanisms, credit guarantees, legal relationship and cooperation norms, and so on (Lu et al., 2016b; Guo et al., 2021). According to institutional theory, these institutional mechanisms can be divided into two categories, namely, informal institutional mechanisms and formal institutional mechanisms. Informal institutional mechanisms are mechanisms formed by people's natural evolution in the process of market transactions, which embody the ethics and practices of the trading market, such as user feedback mechanism, platform supervision mechanism, and payment custody mechanism, which mainly depend on the market itself to play a regulatory role. Formal institutional mechanisms, such as product traceability mechanism and product certification mechanism, are trust guarantee mechanisms established by the state through laws, policies, and other means. These mechanisms are mandatory and violation of them will be punished in the legal sense (Shao and Yin, 2019).

(3) Factors of institutional mechanism affecting consumer trust in the process of buying PAAP

When judging whether the sellers of PAAP are trustworthy, customers will be influenced by informal and formal institutional mechanisms. From the point of view of informal institutional mechanisms, customers' trust in sellers of PAAP is mainly influenced by user feedback mechanism and platform supervision mechanism. Compared with online purchase of ordinary agricultural products, customers' perceived risk of online purchase of PAAP is greater. To reduce the perceived risk, customers will rely more on the evaluations of existing customers to decide whether to trust sellers or not (Cui and Ma, 2018). According to the theory of trust transfer, customers' trust in online shopping platforms can be transferred to the sellers on the platforms (Cheng et al., 2019; Xiao et al., 2019). That is to say, whether the online shopping platform can effectively screen farmers and supervise the trade of PAAP will affect customers' trust in the platform, thus affecting their trust in the sellers on the platform. From the perspective of formal institutional mechanism, product traceability mechanism and product certification mechanism will affect customers' trust. Using modern information technology, a traceability mechanism can realize information traceability of PAAP from production to circulation to consumption, effectively reducing the information asymmetry, thus affecting customers' trust (Yu et al., 2021). Although the poverty-alleviation attribute of agricultural products is difficult to observe, the government's poverty-alleviation certification mechanism for agricultural products can transmit reliable information to customers, thus affecting their trust (Stahl and Strausz, 2017).

In addition, as a stable personality trait, an individual's trust tendency is often used as a moderating variable in research to explore the boundary conditions of trustworthiness factors on consumer trust (Liu et al., 2021). Therefore, we selected the user feedback mechanism and platform supervision mechanism to represent the informal institutional mechanisms, the product traceability mechanism, and the product certification mechanism to represent the formal institutional mechanisms, introduced the individual trust tendency as the moderating variable, and built a research model of the influence of institution-based trust on online purchasing intention of PAAP. Since the effectiveness of institutional mechanisms is a subjective perception of customers, we discussed the influence of four institutional mechanisms on customers' trust from the perspective of consumer perception by referring to existing studies (Huang et al., 2017).

### Research Hypothesis

(1) The impact of the effectiveness of institutional mechanisms on customers' trust

First, the effectiveness of the user feedback mechanism will affect customers' trust. The user feedback mechanism is a large-scale word-of-mouth network artificially built based on the two-way communication ability of the Internet, which mainly includes dynamic evaluation of stores, cumulative sales volume, text and picture comments, and so on. The effectiveness of the user feedback mechanism is the extent to which customers believe that the mechanism can provide accurate and reliable information about the past trading behavior of the seller of PAAP (Wang et al., 2018). On the one hand, due to the non-standardized characteristics of agricultural products, it is difficult for customers to judge the true quality of information about agricultural products they want to buy through the descriptions provided by sellers on the platform before purchase. On the other hand, the authenticity of the “poverty-alleviation” attribute of agricultural products is only known by sellers, but not easily recognized by customers, especially in online transactions where people and goods are separated and experience is lacking. Such differentiation of information possession and cognition will result in unequal status for both parties to the transaction and further strengthen customers' risk perception (Zhou, 2020). Therefore, compared with the online purchase of ordinary agricultural products, customers' perceived risk and uncertainty about the online purchase of PAAP are higher, and it is more necessary to rely on the purchase evaluation of existing customers to reduce perceived risk and uncertainty. Through the user feedback mechanism, customers can obtain relevant information about the seller and the products they sell, and make a basic judgment on whether the seller is trustworthy. From the perspective of signal transmission, the user feedback mechanism can be regarded as an informal signal transmitted by customers. If customers think that this signal is effective, they are more likely to trust this signal and will also trust the seller more (Shao and Yin, 2019). Therefore, hypothesis 1 is put forward as follows:

**Hypothesis 1 (H1)**: The effectiveness of the user feedback mechanism positively affects customers' trust.

Second, the effectiveness of the platform supervision mechanism will affect customers' trust. The platform supervision mechanism consists of the rules, procedures, and systems of an online shopping platform to supervise all transactions by examining trading activities and imposing sanctions on illegal activities (He and Liang, 2018). The effectiveness of the platform supervision mechanism is the degree to which customers think that the mechanism can ensure that the trade of PAAP is implemented as expected. In response to the national policy, Pinduoduo, JD.COM, Suning.cn, and many other platforms have launched special areas for PAAP, where agricultural products are markedly different from ordinary agricultural products. According to the theory of trust transfer, based on the perceived connection between the online shopping platform and the sellers of PAAP on the platform, customers can transfer their trust in the platform to the sellers of PAAP, which has been proved by many scholars (Shao and Yin, 2019). Studies have shown that the degree of customers' trust in online shopping platforms depends on their perception of the effectiveness of platform mechanisms (He and Liang, 2018; Xiao et al., 2019). In other words, if customers can perceive the role of the platform's supervision mechanism in fulfilling the social responsibilities of poverty alleviation, which include distinguishing the true identity of the poverty-alleviation targets, auditing the sellers, and enabling customers to buy real PAAP, they can generate trust in the platform and then generate trust in the sellers of PAAP on the platform. Therefore, hypothesis 2 is put forward as follows:

**Hypothesis 2 (H2)**: The effectiveness of the platform supervision mechanism positively affects customers' trust.

Third, the effectiveness of the product traceability mechanism will affect customers' trust. The product traceability mechanism refers to the systems or procedures used by the agricultural product regulator to track or transform product-related information (Bhutta and Ahmad, 2021). The effectiveness of the product traceability mechanism is the degree to which customers think that this mechanism can reflect the production and circulation information of PAAP. According to the existing research, a traceability mechanism can timely scan, record, and transmit the information of PAAP from production to circulation to consumption to customers, effectively reducing information asymmetry, and thus enhancing customers' trust (Ping et al., 2018; Yu et al., 2021). By scanning the product logo of “One product, one code,” customers can not only learn the quality and safety information of PAAP, to be more assured in consumption, but also obtain the poor households' file card information, to judge the authenticity of PAAP. Therefore, the product traceability mechanism can strengthen the information transmission of PAAP and effectively reduce opportunistic behaviors in the production process of PAAP. If customers think that the product traceability mechanism is effective, they will tend to trust the seller. Therefore, hypothesis 3 is put forward as follows:

**Hypothesis 3 (H3)**: The effectiveness of the product traceability mechanism positively affects customers' trust.

Fourth, the effectiveness of the product certification mechanism will affect customers' trust. The product certification mechanism consists of a series of rules and systems formulated by the government (Brach et al., 2017). Through these rules and systems, it is proven that agricultural products meet the poverty-alleviation standards. The effectiveness of the product certification mechanism refers to the degree of authenticity and reliability of the written assurance that the mechanism gives the PAAP with specific standards to customers. PAAP are agricultural products that are recognized by government departments with the effect of benefiting poverty. According to the existing research, the certification result can be regarded as a formal signal provided by the government to customers. It can convey some unobservable product attribute information to customers, such as the poverty-alleviation attribute of agricultural products (Stahl and Strausz, 2017). When the authentication result is credible, the signal it sends can effectively reduce the consumer's perception of uncertainty and build trust. Brach et al. (2017) pointed out that customers will decide whether to trust certification results based on the credibility of the source of evidence. In the certification process of PAAP, the source of evidence is the certification mechanism, including the certification process, rules, and procedures. When customers perceive that the government's certification mechanism is more effective, they will have more trust in the certification results and thus have more trust in the sellers of PAAP. Therefore, hypothesis 4 is put forward as follows:

**Hypothesis 4 (H4)**: The effectiveness of the product certification mechanism positively affects customers' trust.

(2) The influence of customers' trust on the online purchase intention of PAAP

Customers' trust is an important driving force of online purchasing intention (Yahia et al., 2018). Customers' trust will affect their online purchase intention in the following two ways: First, when customers think the seller is trustworthy, they tend to spend less time and energy searching for the seller's information and spend less cognitive effort when dealing with the seller. The reduction of non-monetary costs can enhance the experience value of customers in the process of purchasing, thus enhancing their online purchasing intention (Fang et al., 2018). Second, the trust allows customers to subjectively exclude the possibility of opportunistic behavior of sellers, thus reducing countless possible outcomes to a more manageable level, which is conducive to reducing customers' perceived risk, thus forming a positive attitude toward trading behavior and generating purchase intention (Agag and El-Masry's, 2017). Studies have shown that customers' trust in sellers is conducive to increasing their willingness to buy products sold by sellers (Silva et al., 2019; Wei et al., 2019; Lăzăroiu et al., 2020). Therefore, when customers believe that the seller of PAAP is trustworthy, they will have fewer potential concerns about the transaction of PAAP, which is conducive to enhancing their willingness to purchase PAAP online. Thus, hypothesis 5 is put forward as follows:

**Hypothesis 5 (H5)**: Customers' trust positively influences their willingness to purchase PAAP online.

(3) The moderating effect of individual trust tendency

The individual trust tendency refers to the degree to which customers are willing to trust others or other things. It is the general tendency to trust others formed by individuals based on long-term life experience (Wang et al., 2015; Lu et al., 2016a). Trust tendency reflects the individual's temperament, and it often needs to be combined with credibility factors to jointly form trust in others, so trust tendency is usually used as a moderate variable to influence the relationship between credibility factors and trust. When customers decide whether or how much to trust the sellers of PAAP, they will look for relevant clues, and the trust tendency will amplify or diminish the role of clues (Choi, 2019). In this study, the institutional mechanisms are important clues for customers to judge whether the sellers of PAAP are credible or not. When customers themselves have a low tendency to trust, they are unwilling to trust others. It is difficult for them to trust the sellers of PAAP even if they perceive the effectiveness of the institutional mechanisms is high. On the contrary, when customers have a high propensity to trust, they are more likely to trust others; the higher the customers perceive the effectiveness of the institutional mechanisms, the higher their trust in the seller of PAAP will be (Liu et al., 2021). Therefore, hypothesis 6 is put forward as follows:

**Hypothesis 6 (H6)**: The individual trust tendency positively moderates the influence of the effectiveness of institutional mechanisms on customers' trust.

**Hypothesis 6a (H6a)**: The individual trust tendency positively moderates the influence of the effectiveness of the user feedback mechanism on customers' trust.

**Hypothesis 6b (H6b)**: The Individual trust tendency positively moderates the influence of the effectiveness of the platform supervision mechanism on customers' trust.

**Hypothesis 6c (H6c)**: The individual trust tendency positively moderates the influence of the effectiveness of the product traceability mechanism on customers' trust.

**Hypothesis 6 (H6d)**: The individual trust tendency positively moderates the influence of the effectiveness of the product certification mechanism on customers' trust.

**Figure 1:**
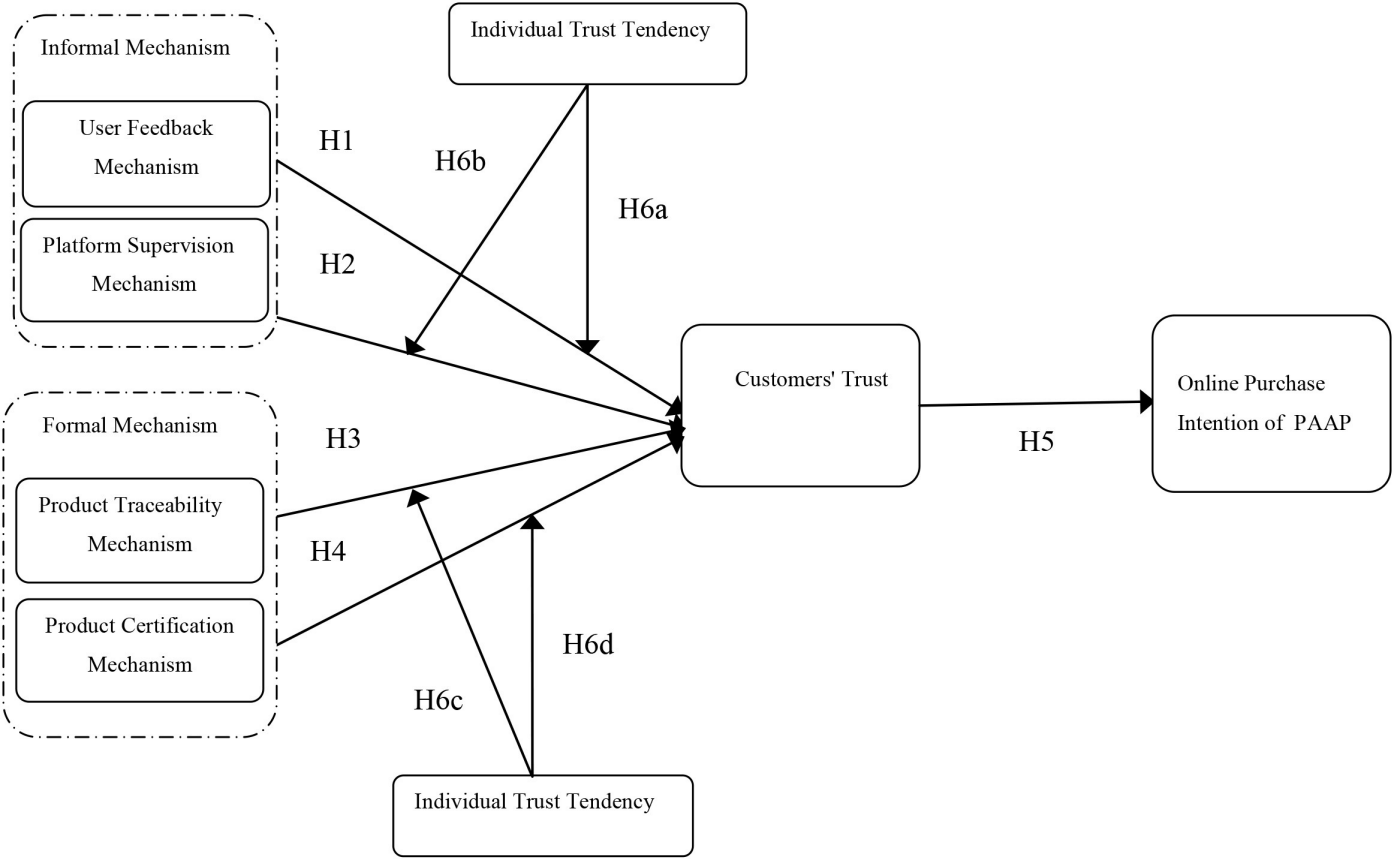
The hypothetical model.

The hypothetical model of this study is shown in Figure 1.

### Research Design and Data Collection

#### Variable Measurement and Questionnaire Design

The questionnaire was made on https://www.wjx.cn, the most used questionnaire design platform in China. To ensure good reliability and validity of the variable measurement, all variables measured in this study refer to the mature scale in existing studies. All the items in the scale are Richter scale 7. The sources of the scale are shown in Table 1.

**Table 1:** Questionnaire design source.

**Variable**	**Reference scale**	**Magnitude**
Effectiveness of user feedback mechanism (UF)	Shao and Yin (2019)	“1” means totally disagree
Effectiveness of platform supervision mechanism (PS)	He and Liang (2018)	“2” means disagree
Effectiveness of product traceability mechanism (PT)	Yu et al. (2021)	“3” means basically disagree
Effectiveness of product certification mechanism (PC)	Stahl and Strausz (2017)	“4” means uncertainty
Customers' trust (CT)	Yahia et al. (2018)	“5” means basically agree
Individual trust tendency (ITT)	Lu et al. (2016a)	“6” means agree
Online Purchase Intention (OPI)	Wei et al. (2019)	“7” means totally agree.

#### Pre-investigation

Before the formal survey, a small pre-survey was conducted in Nanjing, China, and 112 valid questionnaires were collected. In the case of small internal differences in survey objects and a limited number of samples, convenient sampling can obtain data at a low cost. Therefore, in this study, we chose a convenient sampling method to obtain data. SPSS 20.0 software was used to test the validity of the data. The KMO value was 0.823 and passed the Bartlett sphere test (*P* < 0.001), indicating that the data were reliable and exploratory factor analysis could be performed. Exploratory factor analysis results showed that 7 factors were extracted from 27 questions, and the cumulative variance contribution rate was 69.59%. There was no cross-load phenomenon among the questions, and the corresponding relationship with the corresponding factors was consistent with the original scale. The Cronbach's α co-efficients of each factor were all above 0.7, and the Cronbach's α coefficients of each factor would be reduced after the deletion of each item. The above data indicated that each scale had good validity and reliability and could be used for formal research.

#### Formal Investigation

The survey was carried out in the developed regions of eastern China, such as Hangzhou, Shanghai, Nanjing, Suzhou, Changzhou, Wuxi, and so on, which are the main sales areas of PAAP. We collected questionnaire data by combining offline and online methods, using the convenient sampling method. The offline survey was mainly around the commercial plaza, stations, tourist attractions, residential areas, and so on. The investigators randomly invited passersby to fill out questionnaires. The online survey mainly distributed questionnaires through Wechat moments, Wei bo, QQ Space, and other platforms. In the formal investigation, a total of 364 questionnaires were collected. As for the handling of the questionnaire, we set an operational question “Have you ever purchased PAAP through online channels (such as Taobao, Pinduoduo, and wechat mini program)” in the questionnaire, to screen out respondents with online purchasing experience of PAAP. After eliminating invalid questionnaires with too many missed questions, completely consistent answers or obvious regularity, and <1 min of answer time, 328 valid questionnaires were selected as the research sample, with an effective rate of 90.1%. The descriptive statistics of the sample are shown in Table 2.

**Table 2:** Descriptive statistics of the sample.

**Demographic variable**	**Classification**	**The percentage %**	**Demographic variable**	**Classification**	**The percentage %**
Gender	Man	51.61	Education Level	Primary and below	1.82
	Woman	48.39		Junior high school	4.56
Age	Under the age of 18	1.22		Senior high school	29.42
	18–25 years old	16.23		Junior college	23.37
	26–30 years old	16.41		Undergraduate	23.8
	31–40 years old	27.02		Postgraduate and above	17.03
	41–50 years old	17.6	Monthly income	<2,000 yuan	19.45
	51–60 years old	10.22		2,000–5,000 yuan	19.18
	Over 60 years old	11.3		5,001–8,000 yuan	33.4
Marital status	Unmarried	46.78		8,001–11,000 yuan	18.24
	Married	53.22		More than 11,000 yuan	9.73

### Data Analysis and Hypothesis Test

#### Reliability and Validity Tests

(1) Reliability test

In the reliability test of formal sample data, we still used Cronbach's α coefficient to test the internal consistency of each scale. SPSS 20.0 was used to calculate Cronbach's α co-efficients of 7 variables in this study, and the results are shown in Table 3. The Cronbach's α coefficient of each factor was between 0.723 and 0.930, and the Cronbach's α coefficient of the corresponding factor would be reduced after the deletion of each question, indicating that the reliability of the formal questionnaire was good.

**Table 3:** Analysis of the reliability and validity of convergence.

**Construct**	**Measurement items**	**Factor loading**	**AVE**	**CR**	**Cronbach' s α**	**Cronbach' s α(Del)**
Effectiveness of user feedback Mechanism (UF)	UF_1_	0.747^***^	0.698	0.902	0.781	0.757
	UF_2_	0.823^***^				0.700
	UF_3_	0.884**				0.743
	UF_4_	0.880^***^				0.748
Effectiveness of platform supervision mechanism (PS)	PS_1_	0.791^***^	0.641	0.877	0.876	0.846
	PS_2_	0.819^***^				0.837
	PS_3_	0.817^***^				0.842
	PS_4_	0.775^***^				0.840
Effectiveness of product traceability mechanism (PT)	PT_1_	0.823^***^	0.695	0.901	0.864	0.809
	PT_2_	0.844^***^				0.838
	PT_3_	0.796^***^				0.827
	PT_4_	0.869^***^				0.833
Effectiveness of product certification mechanism (PC)	PC_1_	0.798^***^	0.659	0.885	0.851	0.805
	PC_2_	0.752^***^				0.818
	PC_3_	0.834^***^				0.817
	PC_4_	0.860^***^				0.802
customers' trust (CT)	CT_1_	0.866^***^	0.684	0.896	0.894	0.855
	CT_2_	0.826^***^				0.866
	CT_3_	0.854^***^				0.852
	CT_4_	0.757^***^				0.882
Individual trust tendency (ITT)	ITT_1_	0.786^***^	0.659	0.853	0.723	0.658
	ITT_2_	0.835^***^				0.674
	ITT_3_	0.813^***^				0.654
Online purchase intention (OPI)	OPI1	0.820^***^	0.762	0.927	0.930	0.918
	OPI2	0.883^***^				0.910
	OPI3	0.899^***^				0.899
	OPI4	0.887^***^				0.909

*** *P < 0.010*.

(2) Validity test

In this study, AMOS 22.0 was used to test the validity of the formal survey sample data by confirmatory factor analysis. The results showed that χ^2^/df = 1.187 < 5.000, RMSEA = 0.024 < 0.080, GFI = 0.927 > 0.850, CFI = 0.991, NFI = 0.945, RFI = 0.931, IFI = 0.991, TLI = 0.988, all >0.900. All the fitting indexes were better than the fitting reference standard, indicating that the measurement model had a high fitting degree. Based on the results, we used AMOS 22.0 to further test the convergence validity and discriminant validity of the formal survey scale. The convergence validity test results are shown in Table 3. The standardized factor loading coefficients of each item ranged from 0.747 to 0.899, which were all >0.500 and significant at 1% level. The combined reliability (CR) of each scale ranged from 0.853 to 0.927, higher than the standard of 0.700. The average variance extraction (AVE) amount of each scale ranged from 0.641 to 0.762, higher than the standard of 0.500. Therefore, the formal questionnaire had good convergence validity. For the discriminant validity of the scale, the calculation results are shown in Table 4. The diagonal value was the arithmetic square root of the average variance extraction (AVE) amount of each variable, and the value was greater than the absolute value of the correlation coefficient with other latent variables, indicating that the formal questionnaire had good discriminant validity.

**Table 4:** Analysis of the validity of discriminant.

**variable**	**UF**	**PS**	**PT**	**PC**	**CT**	**ITT**	**OPI**
UF	(0.856)						
PS	0.246^***^	(0.851)					
PT	0.203^***^	0.813^***^	(0.834)				
PC	0.256^***^	0.841^***^	0.767^***^	(0.861)			
CT	0.262^***^	0.781^***^	0.708^***^	0.757^***^	(0.827)		
ITT	0.153^***^	0.416^***^	0.333^***^	0.422^***^	0.452^***^	(0.815)	
OPI	0.270^***^	0.686^***^	0.633^***^	0.676^***^	0.736^***^	0.471^***^	(0.873)

*** *Means P < 0.010*.

*Diagonal bracket data are the square root of latent variable AVE value. UF indicates the effectiveness of user feedback mechanism; PS represents the effectiveness of platform supervision mechanism; PT represents the effectiveness of product traceability mechanism; PC represents the effectiveness of product certification mechanism; CT stands for customers' trust; ITT represents individual trust tendency; OPI represents online purchase intention*.

#### Hypothesis Test

In this study, SPSS20.0 was used for multiple regression and hierarchical regression to test the hypothesis. To ensure rigor, demographic factors such as gender, age, and monthly income of the respondents were taken as control variables. Before regression analysis, multiple collinearity tests were conducted on each variable. The results showed that the variance inflation factor (VIF) values of each model were < 10, and the tolerance was >0.1, which indicated that there was no multiple collinearity problem and regression analysis could be conducted.

(1) The main effect test

Model 2 was constructed using the effectiveness of the user feedback mechanism, platform supervision mechanism, product traceability mechanism, and product certification mechanism as independent variables and customers' trust as dependent variables. With customers' trust as the independent variable and online purchase intention as the dependent variable, Model 4 was constructed. Model 1 and Model 3 corresponded to the influence of control variables on customers' trust and online purchase intention, respectively (refer to Table 5).

**Table 5:** The main effect test results.

**Variable**	**Customers' trust**		**Online purchase intention of PAAP**	
**Model**	**Model 1**	**Model 2**	**Model 3**	**Model 4**
Gender	0.102^*^	0.047	0.077	0.004
Age	0.077	−0.043	0.108	0.054
Education level	0.183^***^	0.021	0.184^***^	0.052
Marital status	0.136^**^	−0.070^*^	0.169^***^	0.072
Monthly income	−0.019	0.045	−0.080	−0.067
UF		0.057^*^		
PS		0.395^***^		
PT		0.166^***^		
PC		0.294^***^		
CT				0.715^***^
*R* ^2^	0.073	0.661	0.090	0.564
Δ*R*^2^	0.073	0.588	0.090	0.474
ΔF	5.068^***^	137.924^***^	6.361^***^	349.415^***^

* *Means P < 0.100;*

** *Means P < 0.050;*

*** *Means P < 0.010*.

*UF indicates the effectiveness of the user feedback mechanism; PS represents the effectiveness of the platform supervision mechanism; PT represents the effectiveness of the product traceability mechanism; PC represents the effectiveness of the product certification mechanism; CT stands for customers' trust*.

The results in Table 5 showed that after the effectiveness of the two types of institutional mechanisms was added to the model, the explanatory power of Model 2 was significantly higher than that of Model 1, and the delta *R*^2^ reached 0.588, which indicated that the effectiveness of the two types of institutional mechanisms had a significant impact on customers' trust. Besides, after customers' trust was added to the model, the explanatory power of Model 4 was significantly stronger than that of Model 3, and the delta *R*^2^ reached 0.474, which indicated that customers' trust had a significant impact on the online purchase willingness of PAAP. The results of Model 1 and Model 3 showed that the control variables have different effects on customers' trust and online purchase intention for PAAP.

First, the effectiveness of informal institutional mechanisms positively affected customers' trust (Model 2). Specifically, the effectiveness of the user feedback mechanism and platform supervision mechanism had a significant positive impact on customers' trust. Customers perceived that the more effective the user feedback mechanism was, the more they can trust the sellers of PAAP, assuming H1 was proved (β = 0.057, *P* < 0.100). A study of Didi Taxi customers by Shao and Yin (2019) also showed that feedback mechanisms had a significant positive influence on customers' trust in drivers who provided services on the Didi Taxi platform. When customers perceived that the supervision mechanism of the platform played a greater role in helping the poor people, they were more inclined to trust the sellers of PAAP on the platform, assuming H2 was proved (β = 0.395, *P* < 0.010). Xiao et al. (2019) also proved that when customers believe that an intermediary platform is reliable, they are more likely to believe that the platform has implemented strict rules to manage sellers with whom they cooperate, which helps customers to avoid the opportunistic behavior of sellers and thereby have trust in sellers.

Second, the effectiveness of formal institutional mechanisms positively affected customers' trust (Model 2). In particular, the effectiveness of the product traceability mechanism and product certification mechanism significantly enhanced customers' trust. The more effective the product traceability mechanism was perceived by customers, the more inclined they were to trust the seller of PAAP, assuming H3 was proved (β = 0.166, *P* < 0.010). A study by Yu et al. (2021) on fresh agricultural products also proved that product traceability information had a significant positive effect on customers' trust. The more effective customers perceived product certification mechanisms to be in identifying and helping poor people, the more trust they would have in the sellers of PAAP, assuming H4 was proved (β = 0.294, *P* < 0.010). In sustainable consumption, Brach et al. (2017) found that the perceived credibility of the third-party certification mechanism could effectively reduce customers' risk perception and enhance trust.

Third, the result of Model 4 showed that customers' trust would significantly enhance their online purchase intention of PAAP (β = 0.715, *P* < 0.010), assuming H5 was proved, which was consistent with the research results of most scholars. For example, Yang et al. (2021) conducted a study on customers in Sichuan Province, China, and found that in the context of a live broadcast, customers' trust had a significant positive impact on their online purchase intention of fresh agricultural products. Agag and El-Masry (2017) research on Egyptian consumers also indicated that trust could markedly improve customers' intention to purchase travel online.

Finally, the results of Model 1 and Model 3 showed that the control variables have different effects on customers' trust and online purchase intention of PAAP. Gender significantly affected customers' trust (β = 0.102, *P* < 0.100), indicating that female customers were more likely to trust sellers of PAAP. Gender had a positive effect on the online purchase intention of PAAP, but it was not statistically significant (β = 0.077, *P* > 0.100). Age had a positive influence on customers' trust (β = 0.077, *P* > 0.100) and online purchase intention (β = 0.108, *P* > 0.100) of PAAP, but it was not statistically significant. Education level had a significant positive impact on customers' trust and online purchase willingness of PAAP. It showed that the higher the education level, the easier it was for customers to trust the sellers of PAAP (β = 0.183, *P* < 0.010) and more willing to buy PAAP (β = 0.184, *P* < 0.010). Marital status also significantly positively affected customers' trust and online purchase willingness of PAAP, indicating that married people were more likely to trust the sellers of PAAP (β = 0.136, *P* < 0.050) and were more willing to buy PAAP online (β = 0.169, *P* < 0.010). Monthly income had a negative but insignificant effect on customers' trust (β = −0.019, *P* > 0.100) and online purchase intention (β = −0.080, *P* > 0.100).

(2) The moderating effect test

We used the hierarchical regression method to test the moderating effect of individual trust tendency. The specific method was as follows: The variables were first centralized, and then the interaction terms were obtained by multiplying the independent variables and moderating variables, respectively. Then hierarchical regression analysis was conducted. First, gender, age, education level, marital status, monthly income, effectiveness of user feedback mechanism, effectiveness of platform supervision mechanism, effectiveness of product traceability mechanism, effectiveness of product certification mechanism, and individual trust tendency were considered independent variables, and customers' trust was considered dependent variable in Model 5. Second, based on Model 5, the interactive items of the respective variables and the moderating variables were, respectively, included to construct Models 6, 7, 8, and 9. The results are shown in Table 6. The results of Models 6, 7, and 8 showed that individual trust tendency positively moderated the relationship between the effectiveness of the user feedback mechanism (β = 0.055, *P* < 0.100), platform supervision mechanism (β = 0.055, *P* < 0.100), product traceability mechanism (β = 0.048, *P* < 0.050), and customers' trust, assuming that H6a, H6b, and H6c were verified. The results of Model 9 indicated that the moderating effect of individual trust tendency between the effectiveness of the product certification mechanism and customers' trust was positive but not statistically significant (β =0.078, *P* > 0.100), assuming that H6d was not proved.

**Table 6:** The moderating effect test results.

**Variable**	**Customers' trust**				
**Model**	**Model 5**	**Model 6**	**Model 7**	**Model 8**	**Model 9**
Gender	0.048	0.048	0.050	0.048	0.051
Age	−0.050	−0.060	−0.047	−0.045	−0.041
Education level	0.012	0.010	0.014	0.018	0.018
Marital status	−0.070^*^	−0.074^*^	−0.068^*^	−0.068^*^	−0.065
Monthly income	0.048	0.051	0.048	0.045	0.049
UF	0.051	0.060^*^	0.056	0.054	0.058^*^
PS	0.362^***^	0.365^***^	0.358^***^	0.366^***^	0.365^***^
PT	0.262^***^	0.262^***^	0.266^***^	0.270^***^	0.253^***^
PC	0.178^***^	0.178^***^	0.178^***^	0.163^***^	0.184^***^
ITT	0.130^***^	0.137^***^	0.144^***^	0.141^***^	0.151^***^
UF ^*^ ITT		0.055^*^			
PS ^*^ ITT			0.055^*^		
PT^*^ ITT				0.048^**^	
PC ^*^ ITT					0.078
*R* ^2^	0.675	0.677	0.677	0.677	0.680
Δ*R*^2^	0.602	0.003	0.003	0.002	0.005
ΔF	117.199^***^	2.722^*^	2.689^*^	1.937^**^	5.301^**^

* *Means P < 0.100;*

** *Means P < 0.050;*

*** *Means P < 0.010*.

*UF indicates the effectiveness of the user feedback mechanism; PS represents the effectiveness of the platform supervision mechanism; PT represents the effectiveness of the product traceability mechanism; PC represents the effectiveness of the product certification mechanism; CT stands for customers' trust; ITT stands for individual trust tendency*.

## Discussion on Hypothesis Test Results

Through multiple regression analysis and hierarchical regression analysis, this study verified the relationship between the effectiveness of the user feedback mechanism, platform supervision mechanism, product traceability mechanism, product certification mechanism, and customers' trust and online purchase intention of PAAP, and the moderating effect of individual trust tendency between institutional mechanism effectiveness and consumer trust. The overall results of hypothesis testing in this study are shown in Table 7.

**Table 7:** Summary of hypothesis test results.

**Number**	**Hypothesis**	**Results**
H1	The effectiveness of user feedback mechanism positively affects customers' trust	Proved
H2	The effectiveness of platform supervision mechanism positively affects customers' trust	Proved
H3	The effectiveness of product traceability mechanism positively affects customers' trust	Proved
H4	The effectiveness of product certification mechanism positively affects customers' trust	Proved
H5	Customers' trust positively influences their willingness to purchase PAAP online	Proved
H6a	Individual trust tendency positively moderates the influence of the effectiveness of user feedback mechanism on customers' trust	Proved
H6b	Individual trust tendency positively moderates the influence of the effectiveness of platform supervision mechanism on customers' trust	Proved
H6c	Individual trust tendency positively moderates the influence of the effectiveness of product traceability mechanism on customers' trust	Proved
H6d	Individual trust tendency positively moderates the influence of the effectiveness of product certification mechanism on customers' trust	Not proved

According to the hypothesis test results, most of the hypotheses in this study have passed the test. In other words, the effectiveness of the user feedback mechanism, platform supervision mechanism, product traceability mechanism, and product certification could effectively enhance customers' trust in the seller of PAAP, thus increasing their willingness to purchase PAAP online. Hypotheses H1, H2, H3, H4, and H5 were all proven, which is consistent with Brach et al. (2017); Shao and Yin (2019); Xiao et al. (2019), Yang et al. (2021), and Yu et al. (2021). The results of the comparative analysis verify that the factors that generate trust in other contexts can also have an impact on trust in PAAP transactions and, through trust, on online purchase intentions. In this process, the individual trust tendency played a positive moderating role in the effectiveness of the user feedback mechanism, platform supervision mechanism, product traceability mechanism, and customers' trust. Thus, H6a, H6b, and H6c were verified. However, H6d failed as the individual trust tendency did not play a moderating role between the effectiveness of the product certification mechanism and customers' trust. In this regard, after consulting relevant literature and materials, this study holds that the possible reason is the main body of PAAP certification in China is government departments rather than private institutions. Studies have shown that Chinese urban customers generally have a high degree of trust in the government, which is less affected by the individual trust tendency (Qiu et al., 2007). In February 2022, the world's largest public relations consulting firm Edelman's latest global trust report “Edelman Trust Barometer 2022” also showed that Chinese respondents' trust in their own government reached 91%, ranking first among 28 countries for four consecutive years. Therefore, even though customers themselves had a low trust tendency, when they believed that the government's poverty-alleviation certification mechanism for agricultural products could accurately identify and help poor people, they were willing to believe the government's certification results and then believe the sellers of PAAP.

## Conclusion and Policy Implications

### Conclusion

First, effective informal institutional mechanisms can enhance customers' trust. Under the network environment, sellers can only display the information about PAAP through words, pictures, and videos. Customers cannot know the real information about agricultural products before receiving them, so the experience of others becomes an important basis for customers to judge whether the sellers and the PAAP they sell are credible. The more effective the user feedback mechanism, the more customers can trust the sellers of PAAP. Although the e-commerce platform does not directly participate in the trade of PAAP, it can identify and screen the sellers by formulating rules and procedures and supervise the trading behavior to enhance customers' trust in the sellers of PAAP on the platform. Therefore, the effectiveness of the platform supervision mechanism is conducive to improving customers' trust. Second, effective formal institutional mechanisms are conducive to increasing customers' trust. The product traceability mechanism endows each PAAP with a unique product identification, which can realize the standardization and transparency of the information of PAAP from production, processing, and circulation to consumption and make it easy for customers to grasp the quality and safety of PAAP and the information of poverty-stricken attributes. The decrease in information asymmetry can effectively increase the customers' trust in sellers. The government's poverty-alleviation certification of agricultural products is a kind of commitment and guarantee to customers, which indicates that certified agricultural products can accurately drive poor households to increase income and get rid of poverty. When customers believe that the government's certification mechanism is effective, they can trust the certification results and then trust the sellers of PAAP. Third, trust can significantly enhance customers' willingness to purchase PAAP online. Fourth, individual trust tendency positively moderates the relationship between the effectiveness of the user feedback mechanism, platform supervision mechanism, product traceability mechanism, and customers' trust.

### Policy Implications

First, the sellers of PAAP should pay attention to the establishment and maintenance of their own reputation. They should not only provide real and good quality PAAP to meet the needs of customers, but also pay attention to interaction and communication with customers, and encourage customers who have bought PAAP to express their real experiences and feelings, such as the time efficiency of logistics and the freshness of agricultural products. At the same time, they should actively do a good after-sales service. In view of the problems encountered by customers in the process of purchasing PAAP, it is necessary to remedy them in time.

Second, online shopping platforms should strengthen supervision and do publicity work. On the one hand, it is necessary to shoulder the social responsibility of helping the poor, doing a good job in screening the sellers of PAAP, identifying the sellers' identities, and providing supervision services for the trade of PAAP. At the same time, they should improve the product quality standards on the platforms and strictly control the quality of PAAP. On the other hand, it is essential to increase publicity efforts to enhance customers' perception of the poverty-alleviation image of the platform through advertising and live promotion.

Third, the agricultural product regulator should improve the traceability mechanism of PAAP. They should fully use modern information technology, apply QR code, bar code, and RFID technology to the circulation of PAAP, and timely record the information of the whole process of PAAP from production, processing to transportation and consumption, to realize information sharing and reduce the information asymmetry of customers.

Fourth, the government should enhance the transparency of the certification of PAAP. In the process of identifying PAAP, the government should strengthen the standardization of construction of the certification mechanism for PAAP, improve the transparency of the certification process as well as the certification results, and regularly publish the list of PAAP on the “Social Assistance of China” website. Besides, the government can also encourage customers to actively participate in the supervision process of PAAP and to report false information about PAAP through 12,317 poverty alleviation supervision telephone.

Fifth, the government should increase its efforts to educate the public on the concept of integrity. China's government has a high level of credibility in the public; government-led official media such as “People's Daily,” “CCTV news,” and “Xinhua News Agency” are loved and supported by the general public. Thus, the government should make full use of the influence of these mass media to strengthen the education of public honesty and create a good atmosphere for honest trading through Weibo, WeChat, and other Internet information channels, to enhance the trust tendency of individuals.

### Limitations and Future Research

This study focused on the role of the poverty-alleviation functional attributes of PAAP in enhancing customers' willingness to purchase PAAP online. A theoretical model between the effectiveness of institutional mechanisms, customers' trust, and willingness to purchase PAAP online is constructed, and the research hypotheses are tested through empirical analysis. The findings of the study are conducive to enhancing customers' willingness to purchase PAAP online and promoting sustainable development of poverty alleviation by consumption. At the same time, there are limitations to this study. Through literature review, this study identified four dimensions of institutional factors that affect consumer trust, but there may be other factors that affect consumers' trust in online trading of PAAP, and follow-up research can be further carried out here.

## Data Availability Statement

The original contributions presented in the study are included in the article/Supplementary Material, further inquiries can be directed to the corresponding authors.

## Ethics Statement

Ethical review and approval was not required for the study on human participants in accordance with the local legislation and institutional requirements. Written informed consent for participation was not required for this study in accordance with the national legislation and the institutional requirements. Written informed consent was not obtained from the individual(s) for the publication of any potentially identifiable images or data included in this article.

## Author Contributions

GL: guidance on topic selection, article framework formulation, article writing guidance, and article revision. XW: concept proposal and original paper writing. CY: analyzed and interpreted the data and modified paper. All authors contributed to the article and approved the submitted version.

## Funding

This work was funded by Guangming Li (funder), Youth Project of National Social Science Foundation of China-Research on New Citizens' Cultural Consumption Behavior, Constraints and Guidance Measures (No. 14CGL015) and Lin Liu (funder), General Project of National Social Science Foundation of China-Research on Transition economy, Entrepreneur Social Capital and Enterprise Crisis Management (No. 17BGL021).

## Conflict of Interest

The authors declare that the research was conducted in the absence of any commercial or financial relationships that could be construed as a potential conflict of interest.

## Publisher's Note

All claims expressed in this article are solely those of the authors and do not necessarily represent those of their affiliated organizations, or those of the publisher, the editors and the reviewers. Any product that may be evaluated in this article, or claim that may be made by its manufacturer, is not guaranteed or endorsed by the publisher.

## Footnotes

^1^The Xinhua News Agency. Opinions of the CPC Central Committee and The State Council on comprehensively promoting rural revitalization and accelerating agricultural and rural modernization[DB/OL].http://www.gov.cn/zhengce/2021-02/21/content_5588098.htm.2021-02-21/2021-08-13.

^2^People's Daily Online. poverty-alleviation through consumption will be sustained and sustained. https://author.baidu.com/home?from=bjh_article&app_id=1537195104256385.2021-02-03/2021-09-20.
