# Corrigendum: Research on Improving Online Purchase Intention of Poverty-Alleviation Agricultural Products in China: From the Perspective of Institution-Based Trust

**DOI:** 10.3389/fpsyg.2022.949121

**Published:** 2022-06-24

**Authors:** Guangming Li, Xianghua Wu, Chao Yuan

**Affiliations:** ^1^Business School, Hohai University, Nanjing, China; ^2^The School of Information and Library Science, The University of North Carolina at Chapel Hill, Chapel Hill, NC, United States

**Keywords:** poverty-alleviation by consumption, online purchase intention of poverty-alleviation agricultural products, institution-based trust, helping the poor, trust

In the published article, there was a mistake in the author list as published. The author “Guangming Li” was not included in the published manuscript. The correct author list should be “Guangming Li^1^, Xianghua Wu^1^, Chao Yuan^2^.”

In the published article, there was also a mistake in the Author Contributions statement. The contribution of Guangming Li was missing. The revised statement appears below:

GL: guidance on topic selection, article framework formulation, article writing guidance, and article revision. XW: concept proposal and original paper writing. CY: analyzed and interpreted the data and modified paper. All authors contributed to the article and approved the submitted version.

The authors apologize for these errors and state that this does not change the scientific conclusions of the article in any way. The original article has been updated.

## Publisher's Note

All claims expressed in this article are solely those of the authors and do not necessarily represent those of their affiliated organizations, or those of the publisher, the editors and the reviewers. Any product that may be evaluated in this article, or claim that may be made by its manufacturer, is not guaranteed or endorsed by the publisher.

