# Supplementary material for: Research on Improving Online Purchase Intention of Poverty-Alleviation Agricultural Products in China: From the Perspective of Institution-Based Trust
Source: Front Psychol. 2022 May 12;13:900328. doi: 10.3389/fpsyg.2022.900328 (PMC9133928; doi:10.3389/fpsyg.2022.900328)
Supplement: Supplementary file 1 [file Data_Sheet_1.pdf]

Appendix

Questionnaire on Online Purchase Intention of Poverty Alleviation Agricultural Products

Dear Sir/Madam,

Hello!

Thank you for taking time out of your busy schedule to participate in this survey! We are graduate student in Hohai University, and we are conducting a research on online purchase willingness of poverty-alleviation agricultural products, hoping to get your valuable opinions. According to Article 14 of Chapter III of the Statistics Law of the People's Republic of China, all the information you fill in will be kept strictly confidential and will only be used for academic research. Please fill in it truthfully. Thank you very much for your participation! I wish you all the best!

1. Do you know the poverty alleviation by consumption policy?

No idea at all	I don't know much.	Know something.	A comparative understanding	I know very well.

2. Have you purchased agricultural products for poverty alleviation through online channels (such as Taobao, Pinduoduo, WeChat applet, etc.)?

(Poverty-alleviation agricultural products are agricultural products produced in poverty-stricken areas in the central and western regions of China, certified by the state, which can drive the poor people to increase their incomes and get rid of poverty, covering various categories such as grain, fruits, vegetables, poultry, meat and eggs, etc.)

- Yes (please skip to question 4)
- No purchase

3. What are the main reasons why you didn't buy agricultural products for poverty alleviation?

- Don't know/don't know about poverty alleviation agricultural products.
- No access to poverty alleviation agricultural products
- I don't believe that businesses are selling real poverty alleviation agricultural products.
- Poverty alleviation agricultural products are more expensive than general agricultural products.
- Others ____

4. When was the last time you bought agricultural products for poverty alleviation?

- Within a month
- 1-3 months
- 4-6 months
- 6 months or more

5. What kind of poverty alleviation agricultural products did you buy last time?

- Food
- fruits, vegetables and flowers
- Livestock and poultry products
- Forest products
- Other agricultural and sideline products

6. What was your last platform for purchasing agricultural products for poverty alleviation?

- Taobao
- Tmall
- Pinduoduo
- WeChat applet
- JD.COM
- Suning.cn
- China Social Poverty Alleviation Network
- 832 online sales platform for poverty alleviation
- Central enterprise consumption poverty alleviation app
- Other ____

Before filling out the following questionnaire, please recall the last experience of purchasing poverty alleviation agricultural products on this website, and answer according to your real experience and ideas. Numbers 1-7 represent how much you agree with each statement. Among them, "1" means totally disagree; "2" means disagree; "3" means basically disagree; "4" means uncertainty; "5" means basically agree, "6" means agree and "7" means totally agree. Please fill in the corresponding numbers according to your real experience and feelings.

7. Do you agree with the following statement?

Title	Item	1	2	3	4	5	6	7
	Effectiveness of user feedback mechanism							
UF_1	I think the buyer evaluation of this platform can accurately reflect the quality of poverty-alleviation agricultural products.							
UF_2	I think the buyer evaluation of this platform provides a lot of useful feedback information about the trading history of sellers of poverty-alleviation agricultural products.							
UF_3	I think the buyer evaluation of this platform is effective.							
UF_4	I think the buyer evaluation of this platform is reliable.							
	Effectiveness of platform supervision mechanism							
PS_1	I think the platform is trustworthy.							
PS_2	I think the information of poverty alleviation agricultural products released by this platform is authentic.							
PS_3	I think the platform has corresponding rules and methods, which can ensure that the identity of the sellers of poverty-alleviation agricultural products is authentic.							
PS_4	I think the platform has enough supervision measures to ensure that there are no problems in the trade of poverty-alleviation agricultural products.							
	Effectiveness of product traceability mechanism							
PT_1	I think the traceability QR code of this agricultural product provides a lot of safety information of poverty-alleviation agricultural products.							
PT_2	I can learn about the production and circulation information of poverty-alleviation agricultural products from the traceability QR code of this agricultural product.							
PT_3	The traceability QR code of agricultural products guarantees my right to know about the information of poverty-stricken agricultural products.							
PT_4	The traceability QR code of agricultural products can effectively reduce the information asymmetry of agricultural products.							
	Effectiveness of product certification mechanism							
PC_1	I think the government's certification rules for poverty alleviation agricultural products are fair and credible.							
PC_2	I think the certification process of poverty alleviation agricultural products by the government is fair and credible.							
PC_3	I think the government's certification results for poverty alleviation agricultural products are fair and credible.							
PC_4	I think agricultural products certified by the government will help the poor to increase their income and get rid of poverty.							

Title	Item	1	2	3	4	5	6	7
Consumers' Trust								
CT_1	The platform's sellers of poverty alleviation agricultural products are reliable.							
CT_2	The sellers of poverty alleviation agricultural products on this platform are honest.							
CT_3	The sellers of poverty alleviation agricultural products on this platform are trustworthy.							
CT_4	The sellers of poverty alleviation agricultural products on this platform will keep their promises.							

Title	Item	1	2	3	4	5	6	7
Individual trust tendency								
TT_1	I think most people are trustworthy.							
TT_2	I think people's nature is generally kind.							
TT_3	I usually trust others unless they cheat me.							

Title	Item	1	2	3	4	5	6	7
Online purchase intention								
PI_1	Next time I need agricultural products, I will still buy poverty-alleviation agricultural products on this platform.							
PI_2	I am very willing to browse and buy poverty-alleviation agricultural products on this platform.							
PI_3	I will continue to buy poverty-alleviation agricultural products on this platform.							
PI_4	I would recommend others to buy poverty-alleviation agricultural products on this platform.							

Thank you again for your cooperation! In order to complete the statistical work better and ensure that your questionnaire is effective, I need to collect some basic information of you for research purposes only. Please feel free to fill in it! .

11. Your gender:

Male Female

12. Your age group:

- Under 18 years old
- 18 to 25 years old
- 26 to 30 years old
- 31~40 years old
- 41 ~ 50 years old

13. What is your highest education level?

- Primary school and below
- Junior high school
- High school (including technical secondary school and vocational high school)
- Junior college
- Undergraduate course
- Graduate students and above

14. What is your marital status?

- Married
- Unmarried

15. Your monthly income level is:

- less than 2000 yuan
- 2000-5000 yuan
- 5001-8000 yuan
- 8001-11000 yuan
- 11,000 yuan or more

This is the end of the questionnaire. Thank you again for your participation!
